# Reward-seeking behaviors moderate the association between early life adversity and anhedonia 12 months later

**DOI:** 10.3389/fnbeh.2025.1672103

**Published:** 2025-10-14

**Authors:** Mai-Lan Tran, Uma Rao, Julienne Bower, Andrew Fuligni, Kate Ryan Kuhlman

**Affiliations:** ^1^Department of Psychological Science, University of California, Irvine, CA, United States; ^2^School of Medicine, University of California, Irvine, CA, United States; ^3^Children’s Hospital of Orange County, Orange, CA, United States; ^4^Cousins Center for Psychoneuroimmunology, University of California, Los Angeles, CA, United States

**Keywords:** early life adversity, anhedonia, reward-seeking, adolescents, balloon analogue risk task (BART)

## Abstract

Approximately 20% of adolescents report experiencing anhedonia, conferring high risk for the onset of adolescent depression. Early life adversity (ELA) is associated with anhedonia, and individual differences in reward motivation may inform this association. The current study examined whether reward-seeking behaviors moderated the prospective association between ELA and anhedonia 12-months later among adolescents. During a baseline visit, 74 participants, aged 11–17, completed the Balloon Analogue Risk Task (BART) to measure reward-seeking behaviors via adjusted average balloon pumps. Indeed, participation in the BART has been shown to activate the fronto-striatal neural circuits known to subserve reward-seeking. ELA was assessed continuously via parent-report using a 9-item Adverse Childhood Experiences questionnaire, with scores reflecting cumulative exposures to adversity prior to enrollment; interaction effects were subsequently probed at low, average, and high values for interpretation. At baseline and 12-months later, participants completed the anhedonia subscale within the Reynolds Adolescent Depression Scale 2nd Edition. Adolescents with greater ELA reported more anhedonia 12-months later (*b* = 0.97, *SE* = 0.46, *p* = 0.04), suggesting that ELA confers risk for developing anhedonia. Reward-seeking behavior moderated this association, such that adolescents with more experiences of ELA and low (*b* = 2.35, *SE* = 0.61, *p* < 0.01) and average reward seeking-behavior (*b* = 0.95, *SE* = 0.43, *p* = 0.03), but not high reward-seeking behavior (*b* = −0.45, *SE* = 0.60, *p* = 0.45), were at the greatest risk for increasing severity of anhedonia across the subsequent 12-months. Reward-seeking behaviors may aid in distinguishing which youth with ELA are at risk for depression. Additionally, results from this study may help to inform more specific interventions by increasing reward-seeking behaviors to mitigate the risks of developing anhedonia.

## 1 Introduction

Almost 20% of youth in the United States experience their first major depressive episode between the ages of 10–19 (Bitsko, 2022), making major depressive disorder (MDD) one of the most prevalent mental health disorders among adolescents. The onset of depression during adolescence is a strong predictor of depression in adulthood (Wolitzky-Taylor et al., 2014), highlighting the need for intervention during this developmental period. Anhedonia, defined as the loss of the ability to experience pleasure in previously enjoyable activities, is one of the core symptoms required for a MDD diagnosis (American Psychiatric Association, 2013). Recent research has focused on adolescent anhedonia, as it has been shown to be common among adolescent samples (Thapar et al., 2012; Bennik et al., 2014; Gutkovich, 2014) and a precursor of MDD (Pine et al., 1999; Whitton et al., 2015).

The presence of anhedonia in adolescence is predictive of depression treatment outcomes above and beyond all other depressive symptoms (McMakin et al., 2012). Specifically, greater anhedonia in adolescent depression predicts greater depression severity, increased duration of depressive episodes, fewer depression-free days, elevated suicidal ideation, and poor treatment response to cognitive behavioral therapy (CBT) and antidepressant medication (Gabbay et al., 2015; McMakin et al., 2012). Importantly, anhedonia is considered a cardinal symptom of depression and may represent a particularly proximal pathway linking ELA with later depression onset. Prior reviews suggest that ELA is associated with deficits in reward-related processes, including anhedonia, relative to other depressive symptoms (Pechtel and Pizzagalli, 2011; Pizzagalli, 2014). Thus, while ELA is a distal risk factor for depression more broadly, its close connection to anhedonia suggests that anhedonia may serve as an intermediate mechanism through which ELA confers risk for the development and persistence of depression. Understanding the underlying mechanisms in the development of adolescent anhedonia may provide insight into targeted prevention measures for youth at-risk for developing depression.

Experiences of early life adversity (ELA) may predispose adolescents to anhedonia. Early adverse experiences include a wide range of stressful experiences occurring during childhood and adolescence, such as childhood maltreatment (i.e., physical, sexual, and emotional abuse, physical and emotional neglect) and low socioeconomic status (Felitti et al., 1998). These adverse experiences often involve an individual making significant psychological, behavioral, and neurobiological adaptations in attempts to cope with the experiences that can have implications for negative mental and physical health outcomes (McLaughlin, 2016), including anhedonia (Pechtel and Pizzagalli, 2011). In this context, anhedonia may act as a mechanism to prevent exhaustion from excessive coping behavior, or as a means to secure mental resources by diminishing interest in distractions (van der Gronde et al., 2020). Animal studies in rodents have indicated that ELA, caused by impoverished environments and poor maternal care, leads to anhedonia-like behavior in adolescent rats (i.e., reduced sucrose preference, decreased peer-play) compared to controls (Molet et al., 2016; Bolton et al., 2018). Similarly, adult human studies have shown childhood maltreatment to be associated with greater anhedonia than participants without childhood maltreatment (Fan et al., 2021; Wang P., et al., 2022). Among adolescents, emotional maltreatment, particularly emotional neglect, predicted greater levels and trajectories of anhedonia (Lumley and Harkness, 2007; Cohen et al., 2019). More studies are needed involving adolescent samples to confirm the link between ELA and anhedonia during a developmental period when intervention is most needed.

Individual differences in reward processing behaviors during adolescence may provide insight into which adolescents with ELA are at risk for anhedonia and subsequent pathology. Reward processing is a multidimensional construct consisting of “liking,” “wanting,” and learning” (Berridge and Robinson, 2003). Reward “liking,” or reward attainment, is the experience of pleasure from rewards; reward “wanting,” or reward motivation, is the motivation driving individuals toward rewards; and reward learning refers to behavior guided by previous experiences of rewards and punishments (Berridge and Robinson, 2003). Adolescence is a developmental period during which reward processes are still evolving due to high sensitivity to rewards, and is also a period defined by increases in reward-seeking behaviors that reflects increases in reward motivation (Galvan, 2010). Decreases in reward motivation behaviors such as reward-seeking have implications for anhedonia (Pizzagalli, 2014; Admon and Pizzagalli, 2015; Rømer Thomsen, 2015). Thus, adolescents with ELA and low reward-seeking behaviors may be at the greatest risk for developing anhedonia.

Traditionally, the Balloon Analogue Risk Task (BART; Lejuez et al., 2002) has been widely used to measure risk-taking behaviors among adolescents. The BART is a valid, computerized task designed to simulate real-world risk behavior by balancing the potential for reward and harm. In this task, participants are presented with a balloon and asked to inflate it by clicking a button on the screen. Each click inflates the balloon and adds money to the participant’s temporary winnings. However, each balloon has a variable explosion point. Participants can choose to press “Collect $$$” before the balloon pops, saving their earnings to a permanent bank. If the balloon pops before they collect the money, all earnings for that balloon are lost, and a new balloon is presented. Thus, each pump involves a trade-off between increasing risk and potential reward. The primary outcome of interest is the average number of pumps on unexploded balloons, with higher scores indicating greater risk-taking propensity. However, previous studies have used a similar method as the BART, but to measure reward-seeking behaviors. These studies have used the same ‘button-pressing” mechanism to measure reward-seeking propensities, with non-human animal studies using lever pressing (Salamone et al., 1994; Peciña et al., 2003) and human studies using key-pressing (Aharon et al., 2001; Parsons et al., 2011) to gain rewards. Importantly, unlike a pure risk task where participants can lose previously earned rewards, the BART involves no punishment; if the balloon explodes, the participant simply earns zero rewards on that trial. This design is conceptually closer to effort-based reward tasks such as the effort expenditure for reward task (EEfRT; Treadway et al., 2009), in which each trial has a computable reward value that may or may not be won, but without subtracting from cumulative earnings. This feature supports the use of the BART as a translational measure of reward-seeking motivation rather than risk propensity. Additionally, while the BART is traditionally conceptualized as a measure of risk-taking, neuroimaging studies demonstrate that BART performance engages fronto-striatal regions (e.g., ventral striatum, medial prefrontal cortex) implicated in reward processing (Galvan et al., 2007; Rao et al., 2008). This overlap supports interpreting the task as capturing reward motivation. Distinguishing reward-seeking from risk-taking is then further possible by focusing on adjusted average pumps and statistically controlling for balloon explosions, thereby isolating motivational drive toward reward rather than generalized risk propensity. Thus, this study will explore the use of the BART to measure reward-seeking behaviors among adolescents.

In summary, few studies have examined the association of ELA and anhedonia in adolescents, and to our knowledge, no studies have examined the role of reward-seeking behaviors in this association. Identifying characteristics of adolescents with ELA who are at the greatest risk for anhedonia has implications for prevention and intervention measures targeting reward-seeking behaviors to decrease the risk of developing adolescent anhedonia.

In the present study, the goals were two-fold. First, we examined whether ELA predicted anhedonia 12-months later in adolescents. We hypothesized that ELA would significantly predict anhedonia 12-months later, such that the more ELA participants reported, the more anhedonia that would be reported a year later. Second, we examined whether reward-seeking behaviors moderate the prospective association between ELA and anhedonia 12-months later in adolescents. We hypothesized that reward-seeking behaviors would significantly moderate this association, in which more ELA would predict greater anhedonia 12-months later, specifically among adolescents with lower-reward seeking behaviors.

## 2 Materials and methods

### 2.1 Participants

Participants were 74 adolescents (*M_age_* = 13.86, *SD* = 1.56, Range_age_ = 11–17, 50% female) from a larger study recruited via mass mailing efforts using census record data for households with children ages 12–15 in the Los Angeles and Orange Counties in California, (Kuhlman et al., 2022). Zip codes in the mailing efforts included areas with high rates of poverty and community violence to over-sample for ELA. A more detailed description of recruitment and eligibility criteria can be found in Kuhlman et al. (2022, 2023). Of the 97 participants enrolled in the larger study, 74 (76%) had complete data for the measures in this study. Of the 97 participants enrolled in the larger study, retention at the 12-month follow-up was excellent (99%); however, 21 participants had incomplete BART data and two were missing data on additional variables of interest. Thus, final analytic sample included 74 adolescents with complete data. Of the 74 participants, 43.2% identified as White/Caucasian, 25.7% identified as Hispanic/Latino, 20.3% identified as Asian/Pacific Islander, and 10.8% identified as Black/African American. Regarding annual household income, 12.2% of participant households earned less than $50,000/year, 21.6% of participant households earned $50,000–$100,000/year, and 66.2% of participant households earned more than $100,000/year.

### 2.2 Procedures

Interested parents/guardians of participants completed a phone interview to determine eligibility that included reporting ACEs scores. Once enrolled, participants came into the lab locations at University of California, Los Angeles (UCLA) or University of California, Irvine (UCI), in which the participants and their parents/guardians provided assent and consent. Participants then completed demographic questionnaires and were administered the BART. One year later from the baseline visit, participants completed an exit interview via phone call and completed questionnaires online, which included the anhedonia subscale in the RADS-2. All study procedures were approved by the UCLA and UCI Institutional Review Board.

### 2.3 Measures

*Early Life Adversity*: Early life adversity was measured via parent report using a 9-item checklist adapted from the Adverse Childhood Experiences (ACE) questionnaire (Felitti et al., 1998; Chapman et al., 2004). Parents were asked to respond either “yes” or “no” to whether each of the experiences in the checklist pertained to their child. See Kuhlman et al. (2022, 2023) for the experiences included in the questionnaire. All “yes” responses were summed to form a total ACEs score. Adolescent self-report for ELA was also obtained, which showed moderate convergent validity with parent-reported ACE (Kuhlman et al., 2022). Parent-reported ACE measures are widely used in national data collection efforts, including the National Survey of Child and Adolescent Well-Being (e.g., Helton et al., 2022) and the National Survey of Children’s Health (e.g., Crouch et al., 2019). Moreover, research suggests that parent-report may be particularly reliable when adverse experiences occurred early in development, before the adolescent is able to accurately recall them, or when experiences such as abuse occur within the home environment (Oh et al., 2018). In California, adapted ACE screeners have been integrated into all state-funded pediatric primary care clinics (Purewal et al., 2016; Koita et al., 2018; Thakur et al., 2020), enhancing the clinical and translational relevance of this approach.

*Anhedonia:* Anhedonia was measured using the anhedonia/negative affect subscale within the Reynolds Adolescent Depression Scale-2nd edition (Reynolds, 2004) at two time points: at baseline and 12 months after the baseline visit. The anhedonia/negative affect subscale consisted of 7 reverse-scored items asking how the participant felt over the past month using a 4-point Likert-type scale, with 1 being “almost never” and 4 being “most of the time.” While this subscale captures both reduced pleasure/interest and aspects of negative affect, it provides a validated indicator of anhedonic symptoms within adolescent populations (Blomqvist et al., 2021; Ramos-Vera et al., 2023; Nguyen et al., 2024). Responses were summed to form an anhedonia symptom score that ranged from 1 to 28. The anhedonia subscale demonstrated acceptable reliability in this sample at both baseline and 12-months later, *α* = 0.73 and *α* = 0.75, respectively.

*Reward-seeking behavior:* Participants completed the Balloon Analogue Risk Task (BART) (Lejuez et al., 2002) during the baseline visit to measure reward-seeking behaviors, following the procedure of an earlier within-lab study (see Kuhlman et al., 2023 for a description of the task). Reward-seeking behavior was measured via the adjusted average balloon pump count, or the average balloon pumps on trials where the balloon did not explode. This adjusted value is preferable because the number of pumps is constrained on balloons that exploded, thereby limiting between-participant variability in the absolute averages (Lejuez et al., 2002). We utilized the BART to measure reward-seeking behaviors due to previous non-human animal studies using lever pressing (Salamone et al., 1994; Peciña et al., 2003) and human studies using key-pressing (Aharon et al., 2001; Parsons et al., 2011) to measure reward-seeking behavior. Greater adjusted average balloon pump count reflects greater reward-seeking. Although the BART was originally conceptualized as a measure of risk-taking, our inclusion of balloon explosions as a covariate allowed us to isolate reward-seeking behavior. Thus, our findings specifically reflect motivational drive toward reward rather than generalized risk propensity.

### 2.4 Data analysis

All analyses were conducted in SPSS version 29. ELA was treated as a continuous variable. We used a linear regression to test the association between ELA and anhedonia-12 months later. We then used the PROCESS macro (model 2; Hayes, 2013) to test whether reward-seeking behaviors moderated the association between ELA and anhedonia 12-months later. Interaction effects were probed at −1 SD, 0 SD, and 1 SD values of ELA for interpretation. Exploratory analyses probed interactions where *p* < 0.10. Only results where *p* < 0.05 were considered statistically significant and interpreted. All models accounted for the following covariates: baseline anhedonia, age, sex, race, and risk-taking behaviors via total balloon explosions. Risk-taking behaviors were included as a covariate to align with the traditional purpose of the BART, ensuring that the analyses accounted for potential confounding factors and further isolated the effect of reward-seeking behaviors. Previous studies have also utilized total balloon explosions to index risk-taking behaviors (Lejuez et al., 2002; Kathleen Holmes et al., 2009).

## 3 Results

Descriptive statistics and correlations for participant ACES scores, anhedonia 12-months later, average adjusted pump count, and covariates can be found in Table 1.

**Table 1:** Participant descriptives and correlations (*n* = 74).

Variables	Mean	Standard deviation	Range	1	2	3	4	5	6	7	8
1. ACE	2.08	1.63	0–7	1.00							
2. 12-month Anhedonia	15.86	5.98	7–28	0.17	1.00						
3. Reward-seeking Behavior	25.56	13.85	4.66–78.2	−0.03	−0.08	1.00					
4. Baseline Anhedonia	14.54	5.75	7–28	−0.07	0.30**	−0.15	1.00				
5. Age	13.86	1.56	11–17	−0.10	0.12	0.13	0.20	1.00			
6. Sex	-	-	-	−0.30*	0.07	−0.05	−0.01	0.10	1.00		
7. Race	-	-	-	0.33**	0.03	−0.16	0.12	−0.27*	−0.17	1.00	
8. Risk-taking Behavior	7.07	4.22	1–20	−0.13	−0.03	0.83**	−0.26*	0.11	−0.02	−0.20	1.00

*p < 0.05, **p < 0.01.

Table 2 provides coefficient estimates of anhedonia 12-months later predicted by ELA, reward-seeking behavior, and their interactions. ELA significantly predicted anhedonia 12-months later, such that the more adverse experiences a participant reported, the more severe they reported their anhedonia 12-months later, *b* = 0.97, *SE* = 0.46, *95%CI*[0.05, 1.89], *p* = 0.04 (See Model 1 in Table 2). This association remained significant when adding reward-seeking behavior as a moderator in the model, *b* = 1.11, *SE* = 0.43, *95%CI*[0.26, 1.97], *p* = 0.01 (See Model 2 in Table 2).

**Table 2:** Estimated 12-month anhedonia as predicted by ELA, reward-seeking behavior, and their interactions.

Model	1	2
	*b* (SE)	*b* (SE)
Intercept	5.42 (6.63)	2.57 (6.36)
ACES	0.97 (0.46)*	1.11 (0.43)*
Average adjusted balloon pump count		−0.15 (0.08)
ACES x average adjusted balloon pump count		−0.10 (0.03)*

*p < 0.05, **p < 0.001.

**Figure 1:**
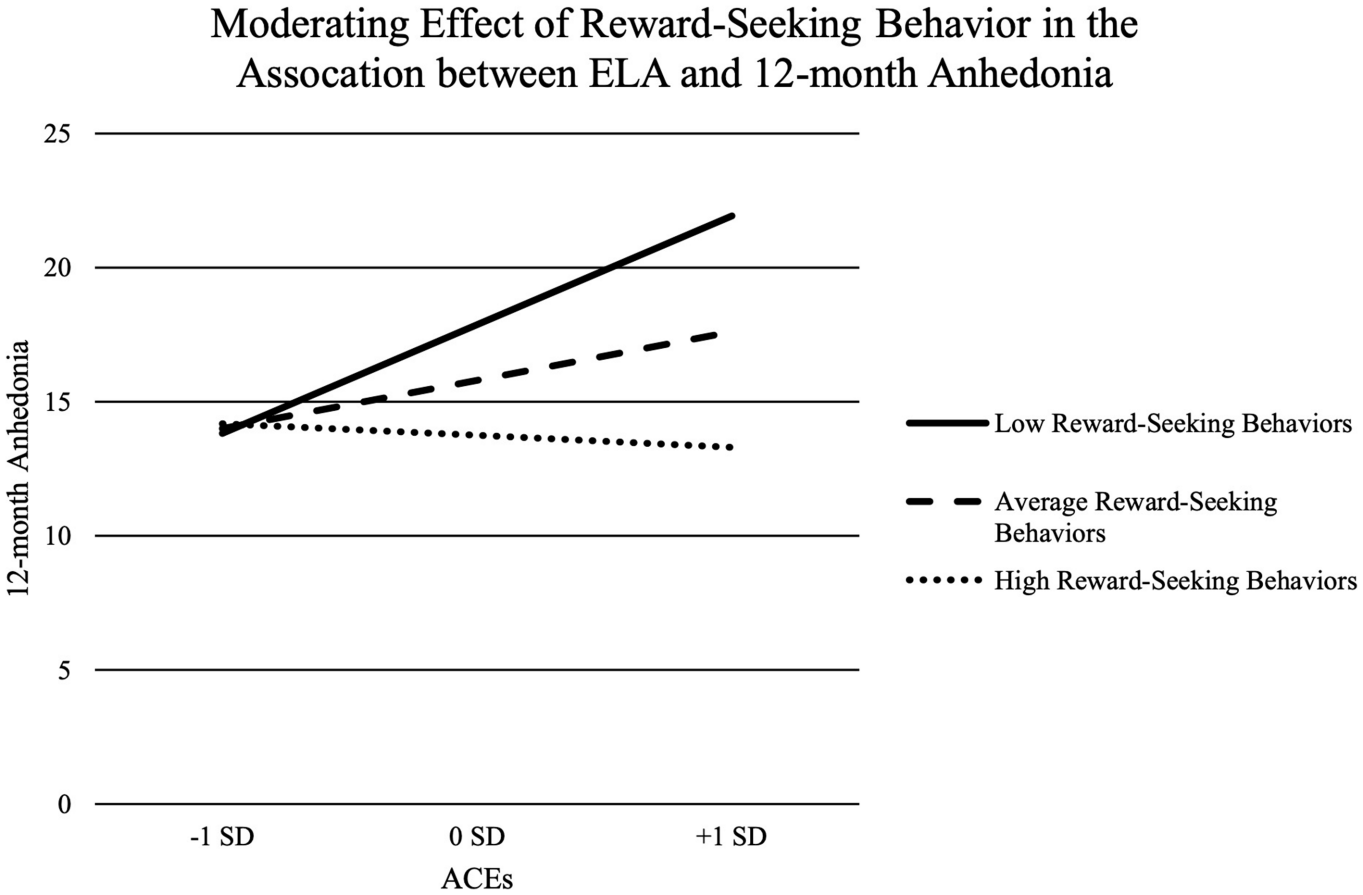
Moderating effect of reward-seeking behavior in the association between ELA and 12-month anhedonia.

Reward-seeking behavior did not significantly predict anhedonia 12-months later, *b* = −0.15, *SE* = 0.08, *95%CI*[−0.31, 0.01], *p* = 0.07 (See Model 2 in Table 2). Reward-seeking behavior significantly moderated the association between ELA and anhedonia 12-months later, *b* = −0.10, *SE* = 0.03, *95%CI*[−0.16, −0.03], *p* < 0.01 (See Model 2 in Table 2 and Figure 1). Specifically, increases in ELA significantly predicted increases in anhedonia 12-months later for adolescents with low (*b* = 2.49, *SE* = 0.60, *95%CI*[1.29, 3.69], *p* < 0.01) and average reward-seeking behavior (*b* = 1.11, *SE* = 0.43, *95%CI*[0.26, 1.97], *p* = 0.01), but not high reward-seeking behavior (*b* = −0.27, *SE* = 0.60, *95%CI*[−1.46, 0.92], *p* = 0.65).

## 4 Discussion

The present study showed that reward-seeking behaviors help to inform the prospective association between ELA and increases in anhedonia 12-months later among adolescents. Specifically, the more ELA a participant had experienced, the more severe anhedonia was reported 12-months later. This was particularly evident in adolescents with low and average, but not high, reward-seeking behaviors, as measured by the BART.

ELA-exposed adolescents with low and average reward-seeking behaviors in this sample showed the greatest increases in anhedonia over the subsequent 12 months. This observation extends our current understanding of which adolescents with ELA may be at the greatest risk for deteriorating mental health in the short term. Importantly, previous research has shown ELA to predict group level risk well, but not individual risk (Baldwin et al., 2021), and limited information exists regarding the individual behavioral differences that predict which individuals with ELA are at the greatest risk for anhedonia. While research has identified individual differences in neural activation (Kirshenbaum et al., 2024), and adversity type (Cohen et al., 2019; Wang X., et al., 2022), no studies have examined the individual differences in behaviors in youth with ELA that predict anhedonia severity. To our knowledge, this study is the first to prospectively link ELA and reward-seeking behaviors to anhedonia 12-months later in a sample of adolescents. Thus, further confirming or identifying other predictive individual differences associated with ELA in adolescents may enrich the current understanding of anhedonia’s multifaceted nature. This would enable a more wholistic approach for detection and prevention programs that could thwart the onset of anhedonia.

Adolescents with high reward-seeking behavior did not show a significant prospective association between ELA and anhedonia. While this nonsignificant pattern may hint at a possible buffering effect, it should be interpreted with caution and requires replication in larger samples. At the same time, the observation raises the possibility that reward-related processes could play a protective role, which aligns with emerging intervention research. Several studies have observed that targeting reward-related processes through intervention can increase reward sensitivity, raise positive affect, and reduce psychopathology symptoms in a clinical sample of adults with depression and anxiety (Craske et al., 2023). For example, Positive Affect Treatment (PAT) resulted in greater improvements in positive affect when compared to a cognitive-behavioral treatment focused on reducing negative affect. At 6-month follow-up, PAT also resulted in higher positive affect, lower negative affect, and lower severity of depressive and anxiety symptoms, stress, and suicidal ideation than the comparison treatment. Whether PAT and other reward-targeting interventions buffer against the development of anhedonia in youth remains unknown but a promising direction for future research.

Our main findings for reward-seeking behaviors remained significant even after considering traditional measures of risk-taking in the BART. This suggests that the effect is specific to reward-seeking, with the BART providing a reliable measure of clinically meaningful reward-seeking behavior in adolescents. Prior research has generally used the BART to assess risk-taking, and has shown the BART to be effective in predicting behaviors related to psychopathology, like increased alcohol and substance use (MacPherson et al., 2010; Fernie et al., 2013; Hanson et al., 2014). Our study extends the BART’s application to evaluating and distinguishing clinically-meaningful reward-seeking behaviors through a short, face-valid task. In particular, this study’s findings on low reward-seeking behaviors in adolescents may reflect the BART’s ability to capture low reward motivation in the real world. Given that adolescence is a critical developmental period involving shifts toward more independence and new experiences (Spear, 2011), reduced reward-motivated behavior (e.g., decreases in the want, effort, or motivation for the reward) has implications for anhedonia and depression. While both measures capture aspects of reward processing, the RADS-2 anhedonia subscale assesses hedonic capacity broadly, whereas BART performance reflects reward motivation, or wanting. Together, they provide converging but non-redundant perspectives on reward processes. However, anhedonia is understood to involve deficits across multiple components of reward processing, including wanting, liking, and learning (Zald and Treadway, 2017). Our study focused on reward motivation (wanting), but did not include behavioral indices of reward liking or learning. Future research should incorporate multiple behavioral measures to more fully capture the multidimensional nature of reward dysfunction.

Reward-seeking behavior in this study was measured using the BART. Individual differences in reward-seeking behavior, as measured by balloon pumps, on this task have been linked to both reactivity and connectivity within the fronto-striatal circuit (Rao et al., 2008; Wang M., et al., 2022), a circuit involved in the reward system (Galvan et al., 2005). In general, adolescents show more activation within the putamen, dorsal lateral prefrontal cortex, frontal lobe, and insula than adults when completing the BART (Wang M., et al., 2022). In adolescents with depression, attenuated functional connectivity between these areas have been linked to anhedonia severity, even after controlling for depression severity (Gabbay et al., 2013). Thus, behavioral measures of reward seeking using the BART may be indexing function or dysfunction within these structures or across this neural circuit. Future studies examining the neural correlates of reward-seeking behaviors measured by the BART and anhedonia could provide a more accurate and sensitive means of detecting anhedonia, particularly in cases where self-report or outward symptoms may not fully capture an individual’s experience.

The results of our study should be considered in the context of several limitations. First, while we over-sampled for ELA, our sample was still relatively small with only 74 adolescents. Second, the 12-month timeline provided only a snapshot of what predicted short-term risk rather than risk for anhedonia across adolescent development, despite adolescents being known to experience years-long periods of depression risk. While 12 months is within the timeframe of extant adolescent depression prevention studies (Stice et al., 2009), studies using longer follow-up periods can examine the trajectory of anhedonia to further inform the long-term impacts of ELA on anhedonia. Third, the use of the BART was limited in measuring reward-seeking behaviors, such that reward-seeking behaviors may not be the only explanation for the link between ELA and anhedonia 12-months later. Reward-seeking behaviors reflected reward motivation, which is just one of the three core components of reward processing “…(wanting, liking, and learning). The present analyses do not reflect behavioral measures of reward liking or learning, which may also play an important role in anhedonia. Future work should incorporate multiple behavioral tasks to capture the full spectrum of reward-related processes.” Fourth, the BART was limited in measuring reward motivation to monetary rewards. Indeed, adolescence is a period marked by heightened sensitivity to a variety of rewards, such as social rewards (Foulkes and Blakemore, 2016), which were not captured by the BART. Future studies should explore reward processing to different rewards to gain a multidisciplinary understanding of altered reward processing among adolescents at-risk for anhedonia. Fifth, it should be noted that the RADS-2 Anhedonia/Negative affect subscale captures both anhedonia and elements of negative affect. Thus, our results may reflect a broader affective dimension, and future work should incorporate additional self-report or behavioral measures of anhedonia to disentangle these constructs. Lastly, adolescents in the sample may have varied in their exposure to common adolescent stressors during the 12-month study period, and this variability could also contribute to risk for anhedonia. The present sample was underpowered to examine the effects of these ongoing stressors, and future research with larger samples should evaluate how ongoing stress interacts with early adversity to shape risk for anhedonia.

To conclude, adolescent anhedonia is a harbinger of MDD (Whitton et al., 2015), emphasizing the need to identify the factors that confer the highest risk for developing anhedonia. ELA is associated with adolescent anhedonia, but not all youth with ELA may go on to develop anhedonia. Thus, identifying exactly which youth with ELA are at the greatest-risk for adolescent anhedonia may provide insight into the types of intervention strategies that may mitigate risk among ELA-exposed adolescents. Findings from our study show that low and average reward-seeking behavior are prospective risk factors in adolescents with ELA for developing anhedonia in the short term.

## Data Availability

The raw data supporting the conclusions of this article will be made available by the authors, without undue reservation.
